# Loss of LasR function leads to decreased repression of *Pseudomonas aeruginosa* PhoB activity at physiological phosphate concentrations

**DOI:** 10.1128/jb.00189-24

**Published:** 2025-05-14

**Authors:** Amy Conaway, Dallas L. Mould, Igor Todorovic, Deborah A. Hogan

**Affiliations:** 1 Department of Microbiology and Immunology, Geisel School of Medicine at Dartmouth 12285, Hanover, New Hampshire, USA; University of California San Francisco, San Francisco, California, USA

**Keywords:** PhoB, LasR, PhoR, quorum sensing, phosphate scavenging, phenazines, CbrB, RhlR, Crc, *Pseudomonas aeruginosa*

## Abstract

The *Pseudomonas aeruginosa* LasR transcription factor plays a role in quorum sensing (QS) across phylogenetically distinct lineages. However, isolates with loss-of-function mutations in *lasR* (LasR– strains) are commonly found in diverse settings, including infections where they are associated with worse clinical outcomes. In LasR– strains, the LasR-regulated transcription factor RhlR can also be stimulated by the activity of the two-component system PhoR-PhoB in low-inorganic phosphate (Pi) conditions. Here, we demonstrate a novel link between LasR and PhoB in which the absence of LasR increases PhoB activity at physiological Pi concentrations and increases the Pi concentration necessary for PhoB inhibition. PhoB activity was also less sensitive to repression by Pi in mutants lacking different QS regulators (RhlR and PqsR) and in mutants lacking genes required for QS-induced phenazine production, suggesting that decreased phenazine production is one reason for increased PhoB activity in LasR– strains. In addition, the CbrA-CbrB two-component system, which can be more active in LasR– strains, was necessary for increased PhoB activity in LasR– strains, and loss of the CbrA-CbrB-controlled translational repressor Crc was sufficient to activate PhoB in LasR+ *P. aeruginosa*. Phenazines and CbrA-CbrB affected PhoB activity independently. The ∆*lasR* mutant also had PhoB-dependent growth advantages in the Pi-deplete medium and increased virulence-associated gene expression at physiological Pi, in part through reactivation of QS. This work suggests PhoR-PhoB activity may contribute to the fitness and virulence of LasR– *P. aeruginosa* and subsequent clinical outcomes.

**IMPORTANCE:**

Loss-of-function mutations in the gene encoding the *Pseudomonas aeruginosa* quorum sensing (QS) regulator LasR occur frequently and are associated with worse clinical outcomes. We have found that LasR– *P. aeruginosa* have elevated PhoB activity at physiological concentrations of inorganic phosphate (Pi). PhoB activity promotes Pi acquisition as well as the expression of QS and virulence-associated genes. Previous work has shown that PhoB induces RhlR, another QS regulator, in a LasR– mutant in low-Pi conditions. Here, we demonstrate a novel relationship wherein LasR represses PhoB activity through the production of phenazines and Crc-mediated translational repression. This work suggests PhoB activity may contribute to the increased virulence of LasR– *P. aeruginosa*.

## INTRODUCTION

*Pseudomonas aeruginosa* is a pernicious pathogen that infects many sites including burns, nonhealing wounds, eyes, and the airways of people with cystic fibrosis (pwCF) or chronic obstructive pulmonary disease. The ability to establish persistent biofilms and acquire critical nutrients, such as phosphorus, in the host environment contributes to *P. aeruginosa* fitness *in vivo*. Phosphate is required for cell membranes, nucleic acids, and metabolic intermediates and is used in many signal transduction pathways. The most accessible form of phosphorus is free inorganic phosphate (Pi), which ranges from 0.8 to 1.4 mM in the serum of healthy adults (1, 2). However, during infection, Pi is restricted by the host as part of nutritional immunity through different mechanisms including the secretion of phosphate-binding proteins (3–5).

Much of host phosphorus is in organic forms that require degradation prior to uptake and utilization by microbes (6). *P. aeruginosa* induces the production of phosphatases, phospholipases, and DNases to access organic phosphate along with high-affinity phosphate transporters in response to low Pi levels via the transcription factor PhoB and its sensor kinase PhoR (7, 8). In *P. aeruginosa*, PhoB also induces the production of specific phenazine small molecules (9–13), which solubilize phosphate from minerals (14). PhoR, which activates PhoB through phosphorylation, is regulated by interactions with the high-affinity Pi transporter PstABC via PhoU such that Pi transport inhibits PhoR activation (15–19). While PhoB is known to positively regulate its own expression (20), data suggest that PhoB also participates in a negative feedback loop (21), presumably to limit intracellular Pi concentrations, as has been described in *Escherichia coli* (22).

The PhoR-PhoB two-component system regulates Pi acquisition and can also play direct roles in virulence regulation in multiple pathogens (8) including *E. coli* (23) and *V. cholerae* (24). In *P. aeruginosa,* cross-regulation between PhoR-PhoB and quorum sensing (QS), which contributes to virulence and biofilm formation (25–27), has been described. *P. aeruginosa* QS is largely controlled by the transcription factors LasR, RhlR, and PqsR, which are active when QS inducers are at sufficient concentrations, i.e. high cell densities or environments with decreased diffusion. While LasR positively regulates both RhlR and PqsR, multiple groups have shown that RhlR and PqsR can be activated in the absence of LasR (28–31). In low-Pi conditions, PhoB induces QS (11–13, 32, 33), and this can occur in a LasR-independent manner (10, 34, 35). One group of virulence factors induced by both PhoB and QS are phenazines, which inhibit immune cell function (36, 37) and kill other microbes (38–40) through oxidative stress. Though PhoB binding sites have been identified upstream of some phenazine biosynthesis genes (13), PhoB induction of phenazine production is largely attributed to increased expression of *rhlR* (10, 13, 32). PhoB regulation of QS suggests low Pi could be a signal for QS remodeling to circumvent reliance on LasR.

*P. aeruginosa* LasR– strains are found in the environment and isolates from both acute and chronic infections, like those in the lungs of pwCF where they make up approximately one-third of clinical isolates (28, 41–46). LasR– isolates are associated with worse disease progression in pwCF (46) and worse lesions from acute ocular infections (43). In laboratory evolution experiments, the increased fitness of *P. aeruginosa* LasR– lineages depends on CbrA-CbrB activity (47, 48), which leads to growth advantages in complex media (29, 47, 49). *P. aeruginosa* CbrA-CbrB is a two-component system that induces expression of the small RNA *crcZ,* which sequesters Crc-Hfq complexes (50, 51). Crc, with Hfq, binds to multiple mRNA targets, many of which encode transporters and catabolic enzymes, and inhibits their translation. CbrA is also required for full *P. aeruginosa* virulence (52). Despite its significance in multiple contexts, the mechanism or cellular cues that modulate CbrA kinase activity, and subsequently Crc-mediated repression, are not yet known.

*P. aeruginosa* virulence regulation is often studied in laboratory media with Pi concentrations that repress PhoR activation of PhoB (e.g., synthetic CF medium (SCFM), 5.1 mM (53); RPMI medium, 5.6 mM (54); Luria–Bertani broth, 6 mM (55); or M9 medium, 64 mM (55)). PhoR-PhoB is generally studied in media with less than 0.5 mM Pi (10, 16, 19, 34). These two extremes in Pi concentrations either strongly repress or strongly induce PhoB activity and thus may not be relevant to our understanding of any contributions of PhoB to *P. aeruginosa* virulence at physiological Pi concentrations (0.8–1.4 mM Pi in healthy serum (1, 2)). Thus, we aimed to elucidate the relationship between LasR and PhoB at physiologically relevant Pi concentrations. In these studies, we show that LasR– isolates and a ∆*lasR* mutant had elevated PhoR-dependent PhoB activity relative to comparator strains with functional LasR at Pi concentrations in the 0.7–1.1 mM range. In contrast, LasR+ and LasR– strains had similar PhoB activity in both low (0.2 mM) and high (10 mM) Pi conditions. Our data demonstrate that a lack of phenazines or reduced Crc activity via CbrA-CbrB led to increased PhoR-PhoB activity and decreased repression of PhoR-PhoB by Pi. PhoB was required for growth in the Pi-depleted medium, and increased PhoB activity in a ∆*lasR* mutant afforded increased growth relative to the wild-type strain. At physiological Pi, PhoB was required for increased expression of multiple virulence factors, including phenazine biosynthetic enzymes and phospholipases, by the ∆*lasR* mutant. This work establishes a novel connection between QS and PhoB wherein LasR represses PhoR-PhoB activity and PhoB is required for increased virulence gene expression in the ∆*lasR* mutant, in part through reactivation of QS. This model for virulence regulation may aid in understanding why *P. aeruginosa* LasR– strains are associated with poor clinical outcomes.

## RESULTS

### PhoB activity is repressed at lower Pi concentrations in *P. aeruginosa* LasR+ strains compared to their LasR– counterparts

**Fig 1:**
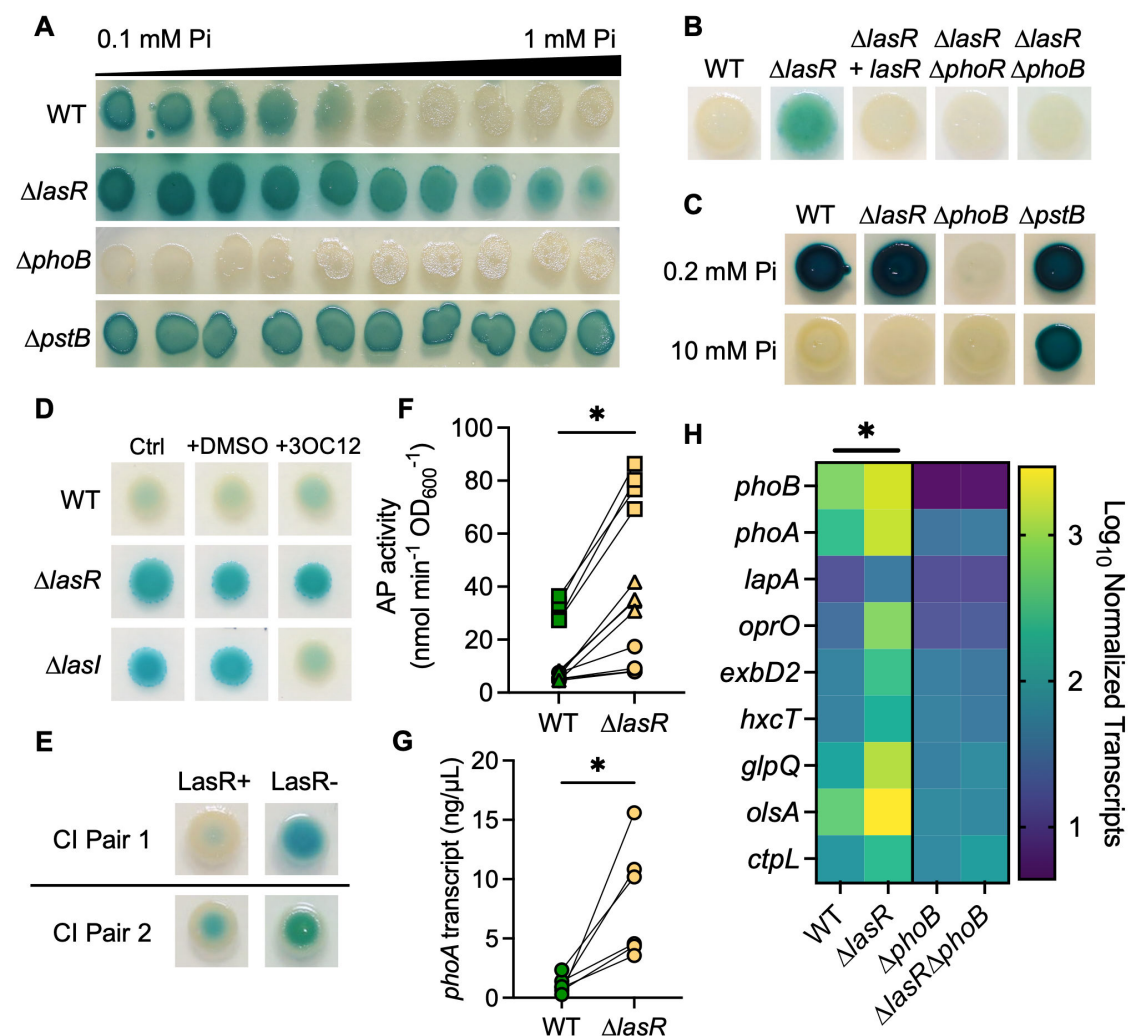
PhoR-PhoB activity is elevated in LasR– *P. aeruginosa* at physiological Pi concentrations. (**A**) *P. aeruginosa* wild type (WT) and ∆*lasR*, ∆*phoB,* and ∆*pstB* mutants were spotted onto MOPS-glucose agar gradient plates with a range of Pi concentrations from 0.1 to 1 mM and 60 µg/mL bromo-4-chloro-3-indolyl phosphate (BCIP) to indicate alkaline phosphatase (AP) activity. (**B**) *P. aeruginosa* strains on MOPS-glucose agar with BCIP and 0.7 mM Pi. (**C**) Colony biofilms on MOPS agar with 0.2% glucose and BCIP with either 0.2 or 10 mM Pi. (**D**) *P. aeruginosa* strains grown as described in B +/– 5 µM 3OC12HSL dissolved in DMSO. (**E**) *P. aeruginosa* clinical isolate (CI) LasR+/LasR– pairs from the sputum of two pwCF grown as described in B. For panels F–H, *P. aeruginosa* was grown as colony biofilms on MOPS-glucose agar with 0.7 mM Pi. (**F**) AP activity in WT and ∆*lasR* was quantified using a colorimetric p-nitrophenylphosphate (PNPP) substrate. Data from replicates collected on the same day have the same shape. Data were analyzed using an unpaired, two-tailed *t*-test (*n* = 12). (**G**) *phoA* transcripts in WT and ∆*lasR* were measured by qRT-PCR on different days and normalized to the housekeeping gene transcript *ppiD*. Data were analyzed using a paired, two-tailed *t*-test (*n* = 6). (**H**) Levels of PhoB-controlled transcripts and *phoB* itself were assessed using NanoString multiplex technology. Nine PhoB-regulated transcripts are shown as normalized counts. Data were analyzed using a two-way ANOVA; there are significant differences between WT-∆*lasR,* WT-∆*phoB*, and ∆*lasR–* ∆*lasR*∆*phoB* (*P* < 0.0001, *n* = 2–3). There are no significant differences between the ∆*phoB-*∆*lasR*∆*phoB* mutants (*P* = 0.46, *n* = 3). Statistical differences for each transcript are available in File S1. For all panels, asterisks denote significance (*P* < 0.05 = *). For panels A–E, similar results were obtained in three replicate experiments; a representative experiment is shown.

Though PhoB regulates virulence factor production across species (8, 23, 24), there are few *in vitro* studies on PhoB activity at Pi concentrations similar to those found in human serum (0.8–1.4 mM Pi (1, 2)). Thus, we sought to determine the Pi concentrations necessary to repress the PhoB activity in *P. aeruginosa* strain PA14. The PhoB activity was monitored by assessing the activity of alkaline phosphatase (AP), which is encoded by the PhoB-regulated gene *phoA* (7), in colonies on agar containing the colorimetric AP substrate bromo-4-chloro-3-indolyl phosphate (BCIP) (56). Using gradient plates with a range of Pi concentrations (0.1 mM–1 mM) in MOPS-glucose agar, we calculated that approximately 0.7 mM Pi repressed AP activity in the wild-type strain (Fig. 1A). Interestingly, a ∆*lasR* mutant had AP activity across the entire gradient, though the activity was reduced at higher Pi concentrations. Consistent with PhoB regulation of AP, the ∆*phoB* mutant had no detectable AP activity at any Pi concentration, and the ∆*pstB* mutant, which has constitutive PhoB activity due to de-repression of PhoR (21), had strong AP production at all Pi concentrations tested. When grown on MOPS-glucose agar with a single concentration of 0.7 mM Pi, the ∆*lasR* mutant colonies had active AP but not the wild-type strain or the ∆*lasR* mutant complemented with *lasR* at the native locus (Fig. 1B). As expected, neither the ∆*lasR*∆*phoR* nor ∆*lasR*∆*phoB* mutants had AP activity. There were no significant differences in the growth of the wild-type and ∆*lasR* strains under these conditions (Fig. S1A and B). Both the ∆*lasR* mutant and the wild-type strain showed AP activity at lower Pi concentrations (< 0.7 mM) (Fig. 1A). We found that at 0.2 mM Pi, a concentration frequently used to study PhoB activity (10, 11, 19), the wild-type and ∆*lasR* strains had similar AP activity (Fig. 1C). Additionally, neither strain showed AP production at 10 mM Pi (Fig. 1C) or on LB agar, which is reported to have 6 mM Pi (55) (Fig. S1C). The ∆*phoB* mutant lacked AP activity even at 0.2 mM Pi, and the ∆*pstB* mutant maintained AP activity at 0.2 mM Pi, 10 mM Pi, and on LB (Fig. 1C; Fig. S1C).

LasR activity is dependent on the autoinducer N-3-oxo-dodecanoyl homoserine lactone (3OC12HSL), which is synthesized by LasR-regulated LasI. Both the ∆*lasR* mutant and the ∆*lasI* mutant had AP activity at 0.7 mM Pi (Fig. 1D). Supplementation of the medium with 5 µM 3OC12HSL reduced the AP activity in the ∆*lasI* mutant relative to the vehicle control or medium alone. Exogenous 3OC12HSL had no effects on AP activity in the wild-type strain or ∆*lasR* mutant. In addition to the strain PA14, we compared the AP activity in two clinical isolate pairs (Fig. 1E) from CF sputum samples wherein one isolate has a loss-of-function (LOF) mutation in *lasR* and the other does not (57). In both cases, the LasR– isolates had increased AP activity relative to their LasR+ counterpart at 0.7 mM Pi. Furthermore, a clinical isolate from a corneal infection, DH2590 (262K in Hammond, *et al*. (43)), which has a mutation resulting in the non-functional LasR^I215S^ variant*,* also expressed AP at 0.7 mM Pi, and AP activity was reduced by replacing the endogenous *lasR* allele with a functional PA14 *lasR* allele at the native locus (Fig. S2A). Another clinical isolate with an LOF mutation in *lasR*, J215 (58), maintained AP activity at even higher Pi concentrations. AP activity in this isolate was also reduced when complemented with the functional PA14 *lasR* allele at the native locus (Fig. S2B).

To quantify AP activity, we utilized the soluble AP substrate p-nitrophenylphosphate (PNPP) and found significantly increased AP activity in the ∆*lasR* mutant compared to the wild-type strain at 0.7 mM Pi (Fig. 1F). Similarly, qRT-PCR analysis of *phoA*, which encodes AP, found significantly higher transcript levels in the ∆*lasR* mutant compared to the wild-type strain (Fig. 1G). When assessing the AP activity of PA14 grown in shaking liquid cultures, we found no increased AP activity in the ∆*lasR* mutant compared to the wild-type strain from 0.1 to 0.7 mM Pi (Fig. S3). In fact, AP activity was significantly decreased in the ∆*lasR* mutant compared to the wild-type strain at 0.3 mM Pi. These data demonstrate that the colony biofilm environment contributes to elevated PhoB activity in LasR– *P. aeruginosa*.

AP activity is a convenient proxy for PhoR-PhoB activity but is encoded by only one of the many genes within the PhoB regulon. To directly assess the levels of transcripts of other genes in the regulon*,* we utilized NanoString multiplex technology as previously published (21). At 0.7 mM Pi, the ∆*lasR* mutant had significantly higher levels of PhoB-regulated transcripts including *phoB* itself and genes encoding phosphatases (*phoA* and *lapA*), a phosphate transporter (*oprO*), a putative TonB transporter (*exbD2*) (59), a type 2 secretion system protein required for secretion of LapA (*hxcT*) (60), a phosphodiesterase (*glpQ*), a low-phosphate ornithine lipid biosynthetic protein (*olsA*) (61), and a Pi chemotaxis protein (*ctpL*) (62). None of these transcripts were significantly different between the ∆*phoB* and ∆*lasR*∆*phoB* mutants (Fig. 1H; File S1).

### Other QS mutants have active PhoB at higher Pi concentrations than wild-type *P. aeruginosa*

**Fig 2:**
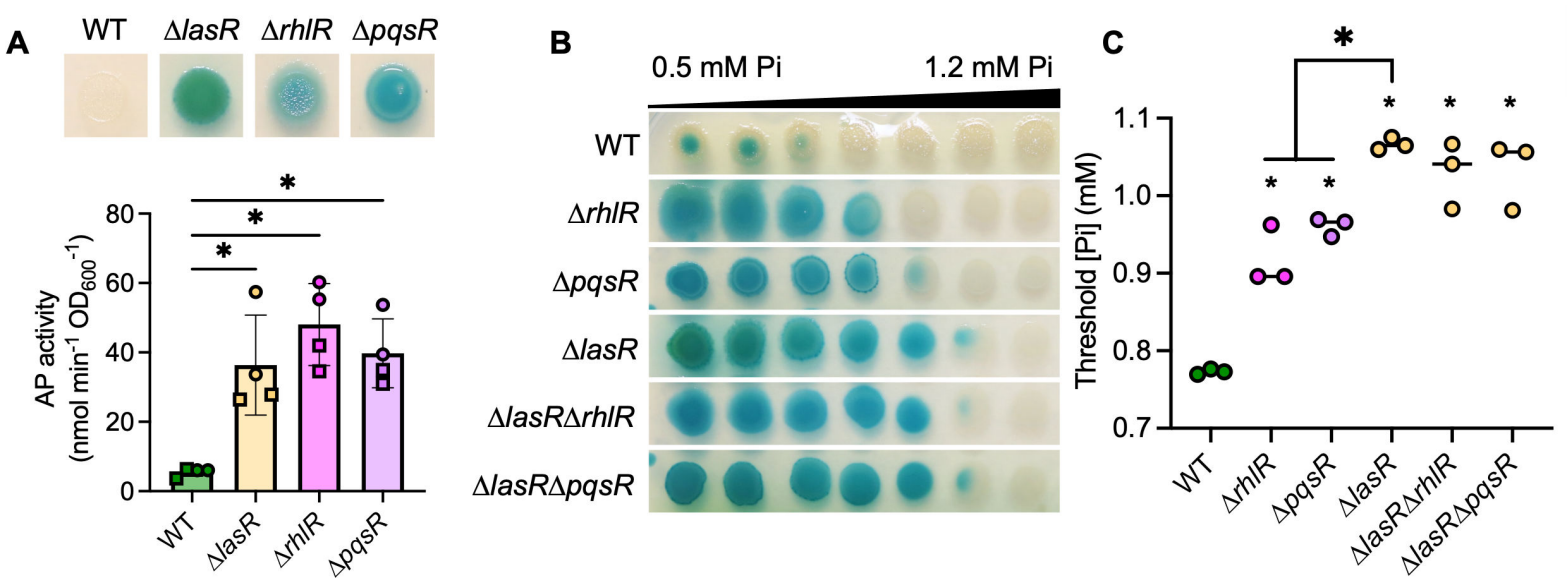
*P. aeruginosa* quorum sensing mutants have active PhoB at higher Pi than the wild type. (**A**) Colony biofilms of WT and the indicated quorum sensing (QS) mutants (∆*lasR*, ∆*rhlR*, and ∆*pqsR*) were grown on MOPS-glucose agar with 0.7 mM Pi and BCIP (top) or on agar without BCIP for analysis of AP activity using the colorimetric PNPP substrate. Data from replicates collected on the same day have the same shape. Data were analyzed by an ordinary one-way ANOVA with Tukey’s multiple comparisons tests (*n* = 4). (**B**) *P. aeruginosa* colony biofilms grown on MOPS-glucose agar with BCIP and 0.5–1.2 mM Pi. (**C**) Threshold Pi was determined as described in the *Methods*. Data were analyzed using a one-way ANOVA and Tukey’s multiple comparisons tests (*n* = 3). Smaller asterisks denote significance from the WT. For all panels, asterisks denote significance (*P* < 0.05 = *).

LasR positively regulates other QS transcription factors including RhlR and PqsR, and some ∆*lasR* mutant phenotypes are due to their decreased activity. However, RhlR and PqsR are capable of inducing QS-controlled genes in the absence of LasR in specific conditions (28, 63). Thus, we assessed the role of RhlR and PqsR in the repression of PhoR-PhoB activity. As shown in Fig. 2A, AP activity was significantly elevated in all QS mutants (∆*lasR,* ∆*rhlR,* and ∆*pqsR*) relative to the wild-type strain at 0.7 mM Pi. Across a gradient plate of 0.5–1.2 mM Pi, AP activity in all QS mutants was inhibited at significantly higher Pi concentrations than the wild-type (Fig. 2B and C). While AP activity in the ∆*rhlR* and ∆*pqsR* strains was inhibited by Pi >0.9 mM, activity in the LasR– strains (∆*lasR,* ∆*lasR*∆*rhlR,* and ∆*lasR*∆*pqsR*) was inhibited by Pi >1 mM (Fig. 2B and C), suggesting a common mechanism for PhoB regulation in QS mutants along with other factors specific to LasR– strains.

### QS-regulated phenazines inhibit PhoB activity

**Fig 3:**
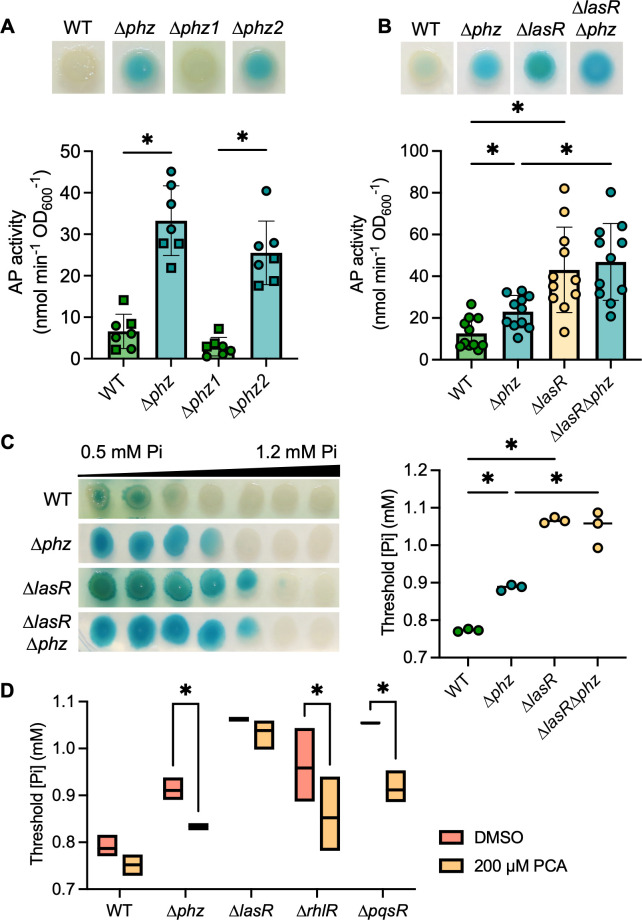
The loss of phenazines promotes PhoB activity. (**A**) *P. aeruginosa* WT and mutants ∆*phz1*, ∆*phz2*, and ∆*phz* (lacking *phz1* and *phz2* operons) grown on MOPS-glucose agar with 0.7 mM Pi and BCIP (top) or on a medium without BCIP for analysis of the AP activity using the colorimetric PNPP substrate. Data from replicates collected on the same day have the same shape (*n* = 7). (**B**) WT and the ∆*phz*, ∆*lasR*, and ∆*lasR*∆*phz* mutants grown as described in A. Data points represent the average of 2–4 colonies analyzed the same day. There were no significant differences between ∆*lasR* and ∆*lasR∆phz* (*P* = 0.92, *n* = 11). Data in A–B were analyzed using a one-way ANOVA and Tukey’s multiple comparisons tests. (**C**) *P. aeruginosa* colony biofilms grown on MOPS-glucose agar with BCIP and 0.5–1.2 mM Pi. The WT and ∆*lasR* mutant are also represented in Fig. 2C. Threshold Pi was determined as described in the *Methods*. Data were analyzed using a one-way ANOVA and Tukey’s multiple comparisons tests (*n* = 3). (**D**) *P. aeruginosa* was grown as described in C with either 200 µM PCA in DMSO or DMSO alone (*n* = 3). Data were analyzed using a matched two-way ANOVA with Sidak’s multiple comparisons tests. For all panels, asterisks denote significance (*P* < 0.05 = *).

As each of the QS transcription factor mutants, ∆*lasR*, ∆*rhlR*, and ∆*pqsR*, produce fewer phenazines than the wild-type strain in late-exponential and early-stationary phase cultures (64, 65), we tested whether the absence of phenazines was sufficient to increase PhoB activity at physiological Pi concentrations. *P. aeruginosa* produces multiple phenazines including phenazine-1-carboxylic acid (PCA) and the PCA derivatives 5-methyl-PCA (5MPCA), pyocyanin (PYO), and phenazine-1-carboxamide (PCN). PCA is synthesized by proteins encoded by two highly similar operons, *phzA1-G1* and *phzA2-G2*. The mutant lacking both *phz* operons (∆*phzA1-G1*∆*phzA2-G2*), referred to as ∆*phz*, had significantly elevated AP activity compared to the wild-type strain at 0.7 mM Pi (Fig. 3A). The ∆*phz1* mutant (∆*phzA1-G1*) was not different from the wild-type strain (Fig. 3A), and deletion of either of the adjacent phenazine-modifying genes required for 5MPCA and PYO biosynthesis, *phzM* or *phzS*, did not lead to changes in AP production at 0.7 mM Pi (Fig. S4A). The additional deletion of *phzH*, which is required for production of PCN, in the ∆*phzHS* and ∆*phzHMS* mutants also had no effect on AP production (Fig. S4B). In contrast, the ∆*phz2* mutant (∆*phzA2-G2*) phenocopied ∆*phz* and had significantly more AP activity than the ∆*phz1* mutant or the wild-type strain (Fig. 3A). We and others have previously published data identifying *phzA2-G2* as the predominant contributor of PCA in colony biofilms (21, 65). Additionally, ∆*phz* required significantly more Pi to suppress PhoB activity than the wild-type strain (Fig. 3C). We found no significant differences in the AP activity at 0.7 mM Pi (Fig. 3B) or the threshold Pi concentration for PhoB activity (Fig. 3C) between the ∆*lasR* and ∆*lasR*∆*phz* mutants, suggesting the ∆*lasR* mutant is already phenazine-deficient in these conditions.

Supplementation of the medium with 200 µM exogenous PCA significantly reduced the Pi threshold for PhoB activation in the ∆*phz* mutant*,* complementing the phenotype (Fig. 3D, images provided in Fig. S5B). Though genetic evidence showed that PCA alone represses PhoB activity at 0.7 mM Pi, we found the other phenazines were also able to reduce the Pi threshold for PhoB activation in some mutants (Fig. S5A). The elevated Pi threshold for PhoB activity in the ∆*phz* mutant was complemented by the addition of PCA, PCN, or PYO. As the ∆*phz* mutant cannot synthesize PCA, adding downstream phenazines to this mutant will not result in off-target changes to the PCA concentration, indicating PCN and PYO can also impact PhoB activity. While there was no significant change in the threshold Pi for PhoB activity when phenazines were added to the ∆*phzHMS* mutant, there was a visible reduction in the overall level of BCIP conversion by PhoA when this mutant was supplemented with any phenazine (Fig. S5B).

Exogenous PCA significantly reduced the Pi threshold in the ∆*rhlR* and ∆*pqsR* mutants but had no significant effect on the wild-type strain or ∆*lasR* mutant (Fig. 3D), demonstrating PCA is not sufficient to restore PhoB sensitivity to Pi in a ∆*lasR* mutant. The wild-type strain was not significantly affected by the addition of any exogenous phenazine. All three phenazines similarly decreased the Pi threshold for PhoB activity in the ∆*rhlR* and ∆*pqsR* mutants (Fig. S5A). Supplementation of PCA did not lead to visible production of blue–green PYO in these mutants, unlike in the ∆*phz* or ∆*lasR* mutants (Fig. S5B). It was previously reported that both RhlR and PqsE positively regulate the expression of *phzM*, which is required to convert PCA to PYO (66). Interestingly, the addition of PCN and PYO each reduced the Pi threshold for PhoB activity in the ∆*lasR* mutant, though PCA had no effect (Fig. S5A). These data further distinguish the regulation of PhoB activity in the ∆*lasR* mutant from that in the other QS mutants.

### CbrA-CbrB-Crc impacts PhoB activity at physiological Pi

**Fig 4:**
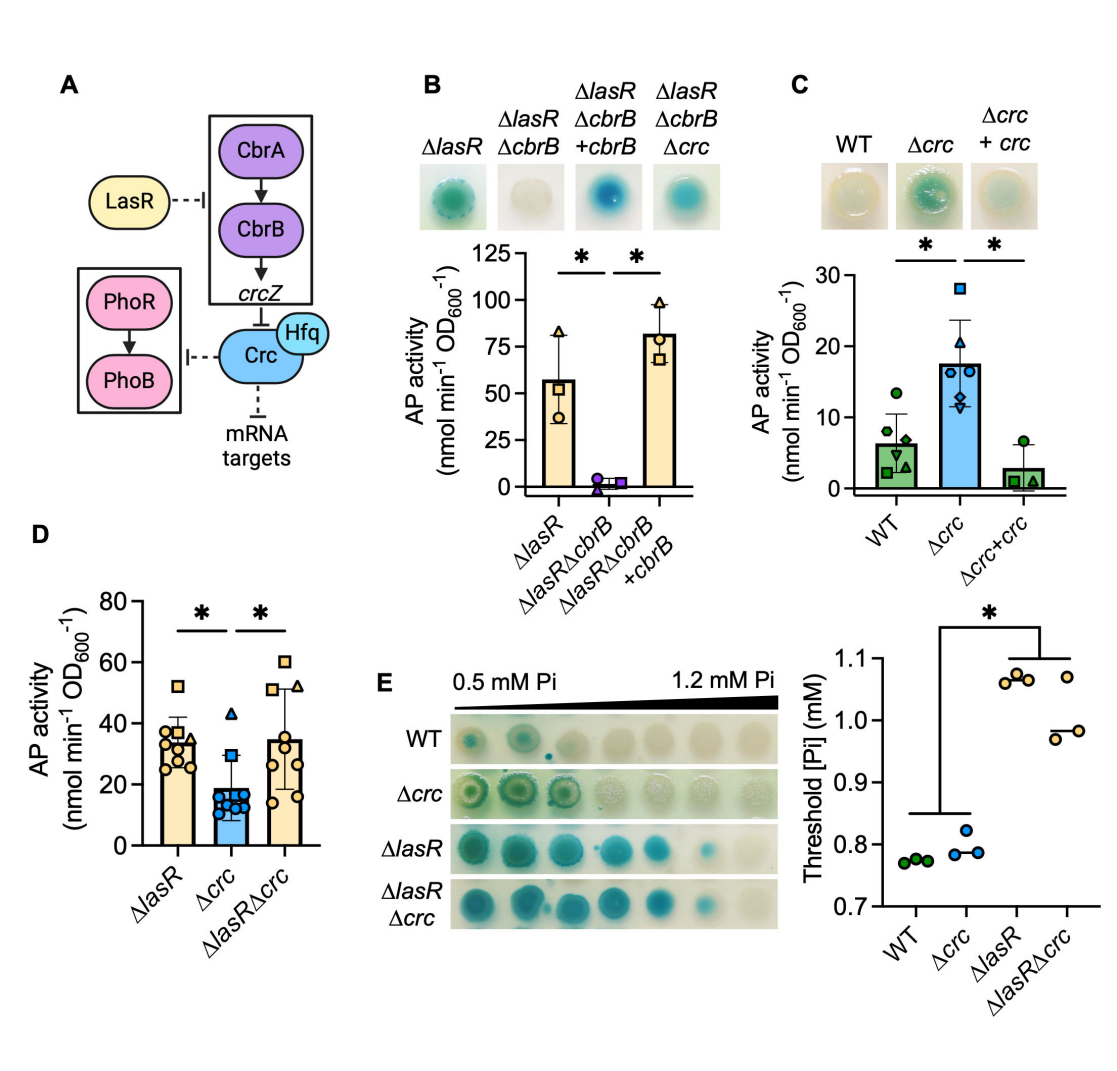
The CbrA-CbrB-Crc pathway promotes PhoB activity in LasR– strains. (**A**) A proposed model of the relationship between the CbrA-CbrB and PhoR-PhoB two-component systems; *crcZ*, a small RNA; Crc, which acts as a translational repressor in complex with Hfq. For figures B–D, AP activity in indicated strains grown on MOPS-glucose agar with 0.7 mM Pi and BCIP (top) or on the medium without BCIP for analysis of AP activity using the colorimetric PNPP substrate. Data from replicates collected on the same day have the same shape. Data were analyzed using a one-way ANOVA and Tukey’s multiple comparisons tests. (**B**) AP activity in ∆*lasR*, ∆*lasR*∆*cbrB*, ∆*lasR*∆*cbrB +cbrB*, and ∆*lasR*∆*cbrB*∆*crc* mutants (*n* = 3). (**C**) AP activity in the WT, ∆*crc* mutant, and its complemented derivative (*n* = 3-6). (**D**) AP activity in ∆*lasR*, ∆*crc*, and ∆*lasR*∆*crc* (*n* = 8). (**E**) *P. aeruginosa* was grown on MOPS-glucose agar with BCIP and a gradient of Pi (0.5–1.2 mM). The WT and ∆*lasR* mutant are also represented in Fig. 2C. The Pi threshold was determined as described in the *Methods*. Data were analyzed using a one-way ANOVA and Tukey’s multiple comparisons tests (*n* = 3). For all panels, asterisks denote significance (*P* < 0.05 = *).

We and others have previously published that LasR– mutants have elevated CbrA-CbrB activity (47, 48), and this leads to growth advantages in multiple conditions. CbrA-CbrB activity leads to relief of Crc-mediated translational repression of diverse mRNA targets, including many that encode catabolic enzymes and transporters (51) (Fig. 4A for model). We hypothesized that CbrA-CbrB activity may mediate increased PhoB activity in the ∆*lasR* mutant as well. We deleted *lasR* from ∆*cbrA* and ∆*phoR* mutants and observed that increased AP activity in a ∆*lasR* mutant required both *phoR* and *cbrA* (Fig. S6A). We also found the ∆*lasR*∆*cbrB* mutant had significantly less AP activity at 0.7 mM Pi (Fig. 4B), though we observed no difference in growth (Fig. S1B). AP production was restored by complementation of *cbrB* at the native locus (Fig. 4B). PhoB activity in both the ∆*cbrB* and ∆*lasR*∆*cbrB* mutants was similarly repressed when Pi >0.3 mM (Fig. S6B). Additionally, AP activity was restored to a ∆*lasR*∆*cbrB* mutant by deletion of *crc* (Fig. 4B), suggesting that the decreased PhoB activity in the ∆*lasR*∆*cbrB* mutant is due to elevated Crc activity (Fig. 4A for pathway). A ∆*crc* single mutant also had significantly elevated AP activity compared to the wild-type and *crc*-complemented strains (Fig. 4C). There was no difference in AP activity between the ∆*lasR* and ∆*lasR*∆*crc* mutants at 0.7 mM Pi, though both are significantly elevated compared to the ∆*crc* mutant (Fig. 4D). Additionally, the ∆*lasR* and ∆*lasR*∆*crc* mutants both showed significantly decreased PhoB sensitivity to Pi relative to the ∆*crc* mutant and the wild-type strain (Fig. 4E).

### CbrA-CbrB-Crc regulates PhoB activity independently of phenazine production

**Fig 5:**
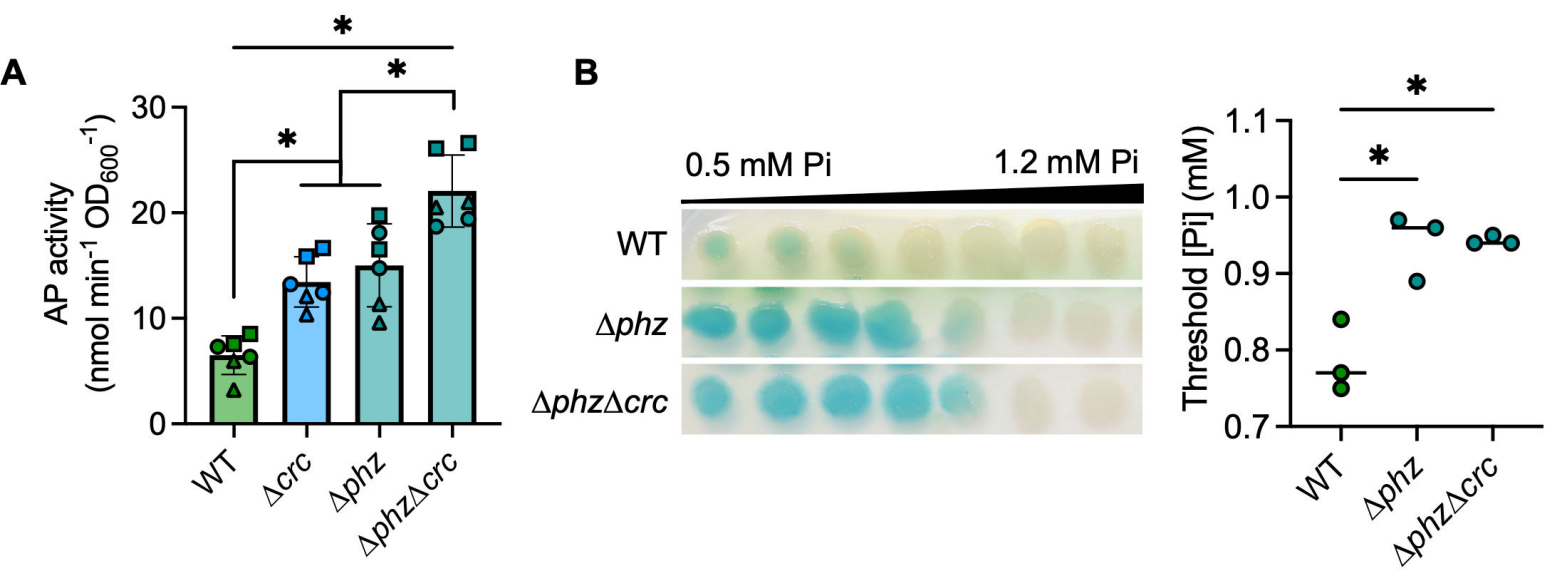
Loss of phenazines and loss of Crc have an additive effect on PhoB activity in LasR+ *P. aeruginosa* at 0.7 mM Pi. (**A**) *P. aeruginosa* colony biofilms grown on MOPS-glucose agar with 0.7 mM Pi for AP activity analysis using a colorimetric PNPP substrate. Shapes indicate data collected on the same day. Data were analyzed using a one-way ANOVA and Tukey’s multiple comparisons tests (*n* = 6). (**B**) *P. aeruginosa* colony biofilms grown on MOPS-glucose agar with BCIP and 0.5–1.2 mM Pi. Threshold Pi was determined as described in the *Methods*. Data were analyzed using a one-way ANOVA and Tukey’s multiple comparisons tests (*n* = 3). For all panels, asterisks denote significance (*P* < 0.05 = *).

Crc-Hfq can repres translation of the transcript encoding the phenazine biosynthesis enzyme PhzM (67). However, we found that AP activity in the ∆*phzM* mutant is not different from the wild-type strain at 0.7 mM Pi (Fig. S4A) and so predicted CbrA-CbrB-Crc affects PhoB activity independently of phenazines. We found the AP activity of a ∆*phz*∆*crc* mutant was significantly increased from the ∆*phz* and ∆*crc* single mutants at 0.7 mM Pi (Fig. 5A). We also found that the addition of 200 µM PCA did not significantly reduce the Pi threshold for PhoB activity in the ∆*crc* mutant (Fig. S5A). However, the addition of PYO did significantly reduce the Pi threshold. The Pi threshold for PhoB activity in both the ∆*phz* and ∆*phz*∆*crc* mutants was significantly greater than that of the wild-type strain and not significantly different from each other (Fig. 5B).

As there were no significant differences in the AP activity at 0.7 mM Pi or the Pi threshold for PhoB activity between the ∆*lasR* mutant and the ∆*lasR*∆*phz* mutant (Fig. 3B and C) or the ∆*lasR*∆*crc* mutant (Fig. 4D and E), we did not expect any additive effects in a ∆*lasR*∆*phz*∆*crc* mutant. At 0.7 mM Pi, there were no significant differences in AP activity between the ∆*lasR,* ∆*lasR*∆*phz,* ∆*lasR*∆*crc,* and ∆*lasR*∆*phz*∆*crc* mutants (Fig. S4C). However, the ∆*lasR*∆*phz*∆*crc* mutant had a significantly lower Pi threshold for PhoB activity than the ∆*lasR,* ∆*lasR*∆*phz,* or ∆*lasR*∆*crc* mutants (Fig. S4D), demonstrating that the loss of both Crc and phenazines increases PhoB sensitivity to Pi in a *lasR* mutant.

### PhoB activity contributes to *P. aeruginosa* ∆*lasR* growth advantages in the Pi-deplete medium

**Fig 6:**
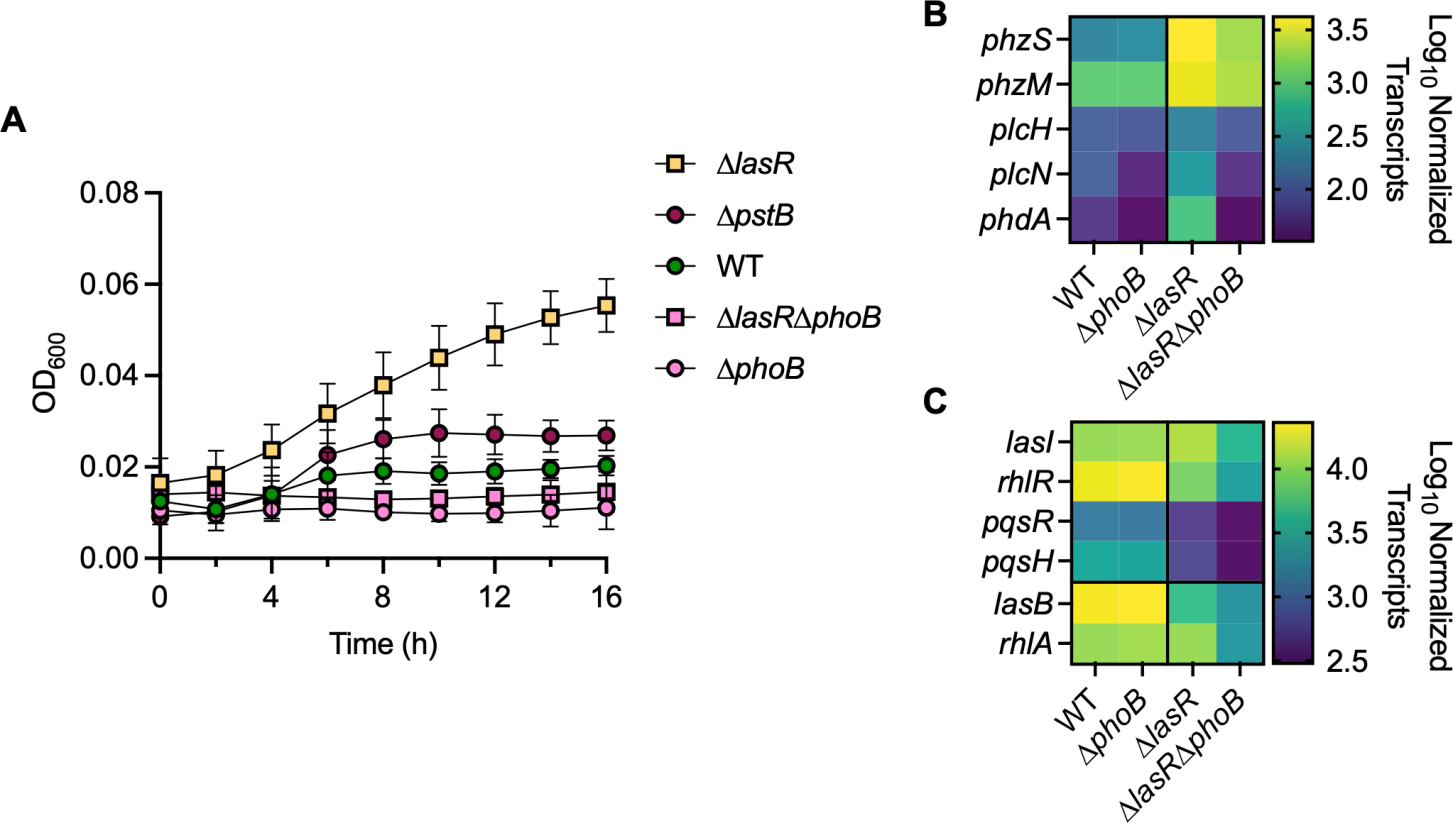
PhoB mediates growth advantages and gene expression in ∆*lasR P. aeruginosa*. (**A**) *P. aeruginosa* WT and ∆*lasR*, ∆*phoB,* ∆*lasR*∆*phoB,* and ∆*pstB* mutants were grown in MOPS-glucose medium with no Pi. Data were analyzed using a two-way ANOVA with Tukey’s multiple comparisons tests comparing each mutant to the WT (*n* = 3). ∆*lasR* is significantly different after 4 hours (*P* < 0.02). ∆*pstB* is significantly different at 10 hours (*P* = 0.037). (**B**) NanoString analysis of PhoB-regulated transcripts shown as log_10_ normalized counts (*n* = 2–3). Transcripts were significantly elevated in ∆*lasR* compared to the WT and ∆*lasR*∆*phoB* (*P* < 0.0001). There was no significant difference between the WT and ∆*phoB* mutant (*P* = 0.28). (**C**) NanoString analysis of QS-regulated transcripts shown as log_10_ normalized counts (*n* = 2–3). There was no significant difference between the WT or ∆*phoB* mutant (*P* = 0.96). Transcripts were significantly elevated in ∆*lasR* compared to ∆*lasR*∆*phoB* (*P* < 0.0001). Complete NanoString data set with statistical analysis of each transcript is available in File S1.

To determine if the elevated PhoB activity in ∆*lasR P. aeruginosa* increased fitness upon depletion of Pi, we grew the wild-type strain, the ∆*lasR* mutant, their respective ∆*phoB* derivatives, and the ∆*pstB* mutant overnight in LB. Cells were then pelleted, the spent LB removed, and then resuspended in the MOPS-glucose medium with no added Pi (Fig. 6A). We proposed that any increased PhoB activity in the LB overnight would allow the cells to increase phosphate stores and later grow better in the Pi-deplete medium. From 4 to 16 hours, the ∆*lasR* mutant grew significantly better than the wild-type strain. The ∆*pstB* mutant, with constitutive PhoR-PhoB activity, grew significantly better than the wild-type strain at 10 hours and maintained a constant but insignificant growth advantage after that. Both the ∆*phoB* and ∆*lasR*∆*phoB* mutants showed minimal growth in the Pi-deplete medium and were not significantly different from each other. These data indicate that PhoB is required for the ∆*lasR* growth advantage, but constitutive PhoB activity is not sufficient to replicate the full advantage.

### PhoB activity mediates the expression of virulence determinants in LasR– *P. aeruginosa,* in part through QS

The NanoString code set used in Fig. 1H included genes that encode proteins associated with phosphate acquisition, virulence, and QS (21). We analyzed the abundance of multiple transcripts encoding virulence-associated proteins, including phenazine biosynthetic enzymes (*phzS* and *phzM*), phospholipases (*plcH* and *plcN*), and an exopolysaccharide matrix regulator (*phdA*) (68) in the wild-type strain and the ∆*lasR,* ∆*phoB,* and ∆*lasR*∆*phoB* mutants. These virulence-associated transcripts were significantly higher in the ∆*lasR* mutant compared to the wild-type strain (Fig. 6B; File S1). Three of these transcripts have also been identified as part of the PhoB regulon (*plcH* (69), *plcN* (9), and *phdA* (59, 70)) while the others have not (*phzM* and *phzS*). However, all five transcripts were significantly lower in the ∆*lasR*∆*phoB* mutant compared to the ∆*lasR* strain, and there were no differences between the ∆*phoB* and ∆*lasR*∆*phoB* mutants. While *plcN* and *phdA* were significantly lower in ∆*phoB* compared to the wild-type strain, *phzM, phzS,* and *plcH* were only PhoB-dependent in the ∆*lasR* mutant at physiological Pi.

As PhoB can induce RhlR expression in low Pi conditions (10, 12, 13, 34, 35), we sought to determine if PhoB contributes to QS at 0.7 mM Pi. We found a significantly higher abundance of transcripts encoding proteins involved in QS regulation (*rhlR* and *pqsR*) and autoinducer synthesis (*lasI* and *pqsH*) as well as transcripts that can be induced by RhlR (*lasB* and *rhlA*) in the ∆*lasR* mutant compared to the ∆*lasR*∆*phoB* mutant (Fig. 6C; File S1). As expected, the combined QS transcripts were significantly less abundant in the ∆*lasR* mutant compared to the wild-type strain. It was unexpected that *lasI,* a direct target of LasR, was not significantly different between the ∆*lasR* mutant and the wild-type strain (File S1). A PhoB binding box has been identified upstream of *lasI,* which could allow PhoB to directly induce expression in the absence of LasR (32). Consistent with the model that PhoB is not active in the wild-type strain at 0.7 mM Pi, there were no significant differences in QS-related transcript abundance between the ∆*phoB* mutant and the wild-type strain.

## DISCUSSION

In this manuscript, we showed that LasR– *P. aeruginosa* laboratory strains and clinical isolates had elevated PhoR- and PhoB-dependent AP activity and increased expression of the PhoB regulon in colony biofilms at 0.7 mM Pi (Fig. 1). We also found that the range of PhoB-permissive Pi concentrations was broader for LasR– *P. aeruginosa*, though PhoB was still repressed at high Pi concentrations. Surface association, decreased nutrient availability, and decreased oxygen are some of the factors in the colony biofilm environment that may contribute to increased PhoB activity. Importantly, Pi concentrations where LasR– *P. aeruginosa* showed more PhoB activity than the wild-type strain are similar to those found in the serum of healthy adults (1, 2).

At 0.7 mM Pi, elevated PhoB activity was common across QS mutants (Fig. 2), and deletion of the *phzA2-G2* operon was sufficient to stimulate PhoB activity (Fig. 3). Unlike the ∆*rhlR* and ∆*pqsR* mutants*,* LasR– *P. aeruginosa* can still produce phenazines in specific contexts (10, 28–30). However, we observed no difference in AP activity between the ∆*lasR* and ∆*lasR*∆*phz* mutants at physiological Pi, suggesting the ∆*lasR* mutant is similarly phenazine-deficient in these conditions (Fig. 3B). The addition of exogenous PCA reduced the Pi threshold for PhoB activity in the ∆*phz,* ∆*rhlR,* and ∆*pqsR* mutants (Fig. 3D). The addition of PCN or PYO reduced the Pi threshold in these mutants as well as the ∆*lasR* mutant (Fig. S5A). It is of note that PhoB activity promotes phenazine production, potentially through direct binding upstream of the *phz* operons (13) though this has been mostly attributed to increased expression of *rhlR* (10, 34, 35). Phenazine production could then reduce PhoB activity, regulating the amount of phosphate inside the cell. As LasR– *P. aeruginosa* maintained PhoB activity at even higher Pi concentrations and was unaffected by PCA, unlike the other QS mutants, we proposed that factors specific to LasR– mutants also contribute to elevated PhoB activity.

LasR– *P. aeruginosa* are more fit than their LasR+ counterparts in many contexts, including diverse media where LasR– *P. aeruginosa* have growth advantages due to increased CbrA-CbrB activity and subsequent relief of Crc-mediated repression (47–49). We showed that CbrA (Fig. S6A) and CbrB (Fig. 4B) were also required for increased PhoB activity in LasR– PA14, and deletion of the CbrB-controlled repressor Crc was sufficient to induce PhoB activity in a LasR+ strain (Fig. 4C). Importantly, the ∆*crc* mutant had less AP activity (Fig. 4D) and was more sensitive to PhoB inhibition by Pi (Fig. 4E) than the ∆*lasR* mutant. These data demonstrate that Crc– *P. aeruginosa* do not entirely phenocopy LasR– strains.

Crc-Hfq is known to repress translation of *phzM,* thus limiting the production of 5MPCA and PYO (67). However, there is no evidence of direct Crc regulation of PCA, and the supplementation of exogenous PCA had no effect on the Pi threshold of a ∆*crc* mutant (Fig. S5A). We also found that complementing the J215 clinical isolate with a functional PA14 *lasR* allele reduced the AP activity at 1 mM Pi but not 0.7 mM Pi (Fig. S2B). J215 has LOF mutations in both *lasR* and *rhlI,* and while complementation of *lasR* would restore CbrA-CbrB activity, RhlI is still required for phenazine production. These data strongly suggested independent roles for phenazines and Crc in repressing PhoB activity. We demonstrated that PhoB activity was further increased in the ∆*phz*∆*crc* mutant relative to either single mutant (Fig. 5A). However, the ∆*lasR*∆*phz*∆*crc* mutant was more sensitive to PhoB repression by Pi than any double mutant (Fig. S4D). Taken together, these data indicate Crc and phenazines independently suppress PhoB activity. Additionally, the loss of both phenazines and Crc-mediated repression can have a deleterious effect in a LasR– strain.

There are multiple possible mechanisms by which LasR activity, through phenazines and Crc, could repress PhoB activity at physiological Pi concentrations. There is evidence that PhoB can be spontaneously phosphorylated by acetyl phosphate *in vitro* (71), but the concentrations required for this reaction suggest it is unlikely to occur inside cells (72). Therefore, we expect changes in PhoB activity are mediated by its sensor kinase PhoR. PhoR activity is suppressed by interactions with the Pi transporter PstABC and PhoU (15–19). Thus, changes in the expression of these proteins could alter the PhoR-PhoB activity. We observed no differences in the transcript abundance of *phoU* or *pstA* between the wild-type strain and ∆*lasR* mutant (File S1), and so this model is unlikely. However, other changes in Pi transport into the cell could still contribute to differences in PhoB activity. PhoR has been shown to interact with PhoU through a Per-Arnt-Sim (PAS) domain (18), which in other proteins can modify kinase domain activity by binding a ligand or promoting dimerization (73). Crc is an important tool for regulating metabolism through translational regulation of transporters and catabolic enzymes. PCA, PCN, and PYO can all act as alternative electron acceptors in various contexts and so modify the metabolic state of the cell (74, 75). Thus, we propose that changes to the metabolic state in phenazine-deficient cells or cells with low Crc activity may stimulate PhoR activity through its PAS domain.

**Fig 7:**
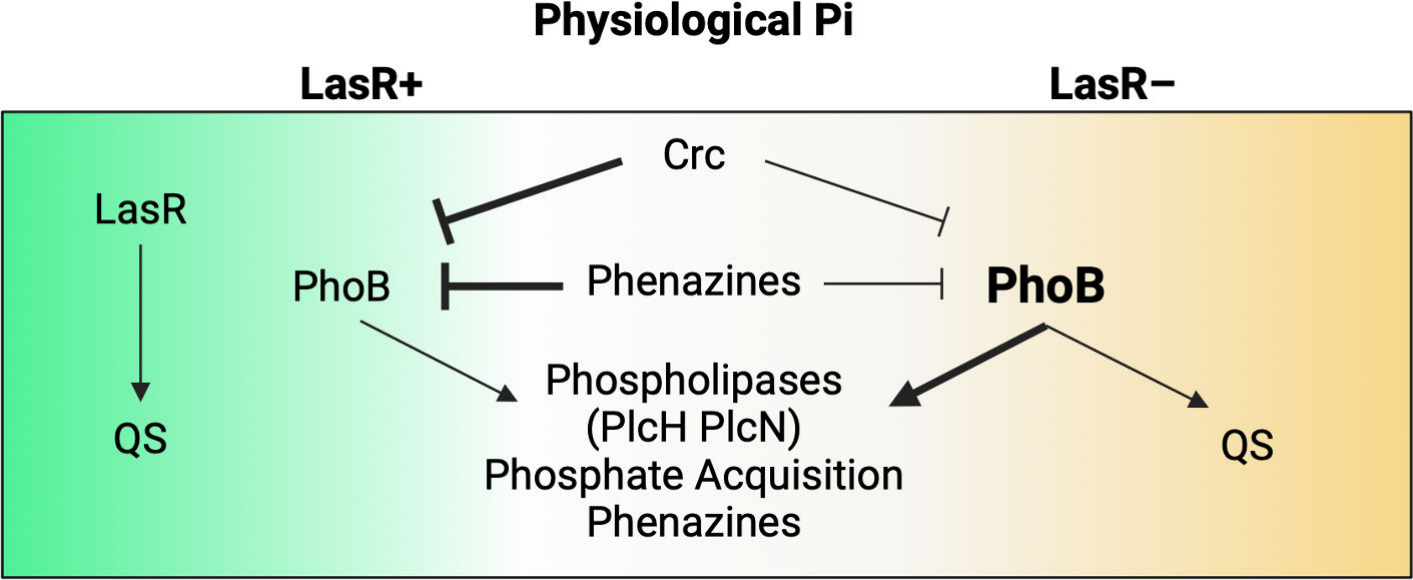
Model of *P. aeruginosa* PhoB activity regulation at physiological Pi. LasR+ *P. aeruginosa* have increased repression of PhoB by Crc and phenazines at physiological Pi. LasR activity contributes to the expression of RhlR and QS activity. LasR– *P. aeruginosa* have increased PhoB activity, leading to the expression of RhlR and QS activity as well as phospholipases at physiological Pi.

We have developed a model wherein LasR– *P. aeruginosa* have elevated PhoB activity at physiological Pi due to decreased phenazine production and decreased Crc-mediated repression (Fig. 7). Increased PhoB activity allows LasR– *P. aeruginosa* to maintain QS signaling and increase the expression of genes critical to survival and virulence. We observed that PhoB was required for improved growth of the ∆*lasR* mutant in the Pi-deplete medium (Fig. 6A). As phosphate accessibility can be transient inside the host, elevated PhoB activity and increased metabolic flexibility through CbrA-CbrB may allow cells to increase the internal stores of polyphosphate for future use during low Pi stress. In addition to regulating phosphate acquisition, PhoB can promote the expression of QS genes directly (32) or through RhlR (12, 13, 35), allowing cells to circumvent reliance on LasR as a QS regulator in low-Pi conditions (10, 34). Induction of RhlR activity by PhoB can then lead to PYO production, swarming motility, and cytotoxicity (10, 12, 13, 34, 35). Our data support and expand upon this existing model by showing QS gene expression is PhoB-dependent in a ∆*lasR* mutant but not the wild-type strain at physiological Pi (Fig. 6C). We also demonstrated that the increased expression of virulence-associated genes in a ∆*lasR* mutant is PhoB-dependent at physiological Pi (Fig. 6B). These genes included phenazine biosynthetic enzymes and phospholipases like PlcH. PlcH is known to degrade airway surfactant, and we have previously shown that its expression leads to a decline in lung function (76).

Importantly, we have shown increased PhoB activity in LasR– *P. aeruginosa* compared to their LasR+ counterparts in colony biofilms, and this has not been reported previously. The relatively low (0.4–0.5 mM) and high (4–4.5 mM) Pi concentrations used in the past likely hindered the observation of increased PhoB activity in LasR– *P. aeruginosa* at physiological Pi and demonstrate the utility of gradient agar plates. The previously reported PhoB-driven reorganization of QS may occur in LasR– *P. aeruginosa* under conditions that otherwise repress PhoR-PhoB activity in LasR+ strains, particularly in surface-associated populations. Understanding LasR– *P. aeruginosa* virulence in physiologically relevant conditions is critical as LasR– isolates are frequently found in both acute and chronic infections. These findings may also be relevant to *P. aeruginosa* in settings that do not induce QS regulation (i.e. low density or high diffusion). The increased PhoB-mediated QS and virulence gene expression in LasR– *P. aeruginosa* may contribute to their association with worse outcomes (43, 46).

## MATERIALS AND METHODS

### Strains and growth conditions

Bacterial strains and plasmids used in this study are listed in Table S1. Bacteria were streaked from frozen stocks onto lysogeny broth (LB) with 1.5% agar (77). Planktonic cultures were grown in 5 mL LB medium in 18 mm borosilicate glass tubes on a roller drum at 37°C.

### Construction of in-frame deletion and complementation plasmids

Construction of in-frame deletion and complementation constructs was performed using yeast cloning techniques in *Saccharomyces cerevisiae* as previously described (77) or with the NEBuilder HiFi DNA Assembly Cloning Kit (NEB #E5520S). Constructs for in-frame deletion of most or all of the coding sequence and chromosomal complementation were made using the allelic replacement vector pMQ30 with 1 kb homology on either side of the gene to be deleted or complemented (78). For all complements, the gene under its native promoter was reconstituted. Plasmids were purified from yeast using Zymoprep Yeast Plasmid Miniprep II according to the manufacturer’s protocol and transformed into *E. coli* strain S17/λpir by electroporation. Plasmids were introduced into *P. aeruginosa* by conjugation, and recombinants were obtained using sucrose counter-selection. Genotypes were screened by PCR, and plasmid constructs were confirmed by sequencing.

### Quantifying growth on MOPS-glucose agar

*P. aeruginosa* was grown overnight in 5 mL LB medium. 5 µL of the overnight culture was spotted onto MOPS-glucose agar with 0.7 mM Pi and incubated at 37°C for 16 hours. The spots were then cored using the broad end of a P1000 pipette tip and the agar cores shaken into 1 mL of PBS + 0.01% Triton-X and disrupted for 5 minutes using a Scientific Industries Genie Disrupter. 100 µL of the cell suspension was serially diluted, and 5 µL of each dilution was spotted on LB agar and incubated for 16–24 hours at 37°C. CFU/mL was calculated from the colony-forming units on this plate.

### Alkaline phosphatase activity assessment

Agar plates were made as previously described (21) with MOPS minimal medium (79) with 20 mM glucose, 15 g/L agar (referred to here as MOPS-glucose agar), 0.7 mM K_2_HPO_4_/KH_2_PO_4_, and 60 µg/mL BCIP (Sigma Aldrich #11383221001). Overnight cultures of *P. aeruginosa* were grown in LB, normalized to an OD_600_ of 1, and then 5 µL was spotted on MOPS-glucose agar and incubated at 37°C for 16–24 hours. Plates with a gradient of phosphate were made as previously described (21) based on the methodology for pH gradient plates (80). First, 35 mL of molten MOPS-glucose agar at one end of the gradient was pipetted into a 10 cm square Petri dish (Corning #BP124-05) that rested in a custom 3D-printed prop that held the plate slanted at a 30° angle. Once the bottom layer had solidified, the plate was laid flat, and 35 mL of molten medium agar containing the second desired concentration of the gradient was poured atop. For each strain, 5 µL spots were plated at consistent intervals across the plate using a grid. The order of strains on the plate was changed for each replicate. Representative images in each figure are shown from the same replicate.

### Calculating alkaline phosphatase enzymatic activity

A colorimetric assay using the 1-Step p-nitrophenyl phosphate (PNPP) substrate (Thermo Scientific #37621) was used to quantify alkaline phosphatase activity in colonies grown on MOPS-glucose agar plates with a single concentration of Pi and no added BCIP using the inoculation regime described above. After 12 hours of incubation, each colony was gently scraped from the agar with a pipette tip and resuspended in 100 µL 10 mM Tris-HCL buffer at pH 8. 50 µL of the cell suspension was mixed with 50 µL of room temperature 1-step PNPP solution (PNPP) or 50 µL of Tris-HCL buffer at pH 8 (NoPNPP) and incubated in the dark at 30°C for 30–60 minutes. A_405_ and A_600_ were recorded in 15–30 minute intervals. The AP activity was calculated using the equation 1000 x (∆A_405_/(time (min) x A_600_)), where ∆A_405_ = (A_405_PNPP – A_405_NoPNPP)t_60_ – (A_405_PNPP – A_405_NoPNPP)t_0_.

### Calculating threshold Pi concentration

*P. aeruginosa* was grown on MOPS-glucose agar with BCIP and a gradient of Pi as described above. When phenazines were included, 9 mM stocks of each phenazine in DMSO were prepared and then added to the media to a final concentration of 200 µM. An equal volume of DMSO was added as a vehicle control. The Pi threshold for PhoB activity was calculated using the equation ((Pi_max_ – Pi_min_)/Plate length) x (BCIP length) + Pi_min,_ where Plate length is the length of the entire plate and BCIP length is the distance from the low-concentration edge of the plate to the point where BCIP conversion to the blue pigment stopped.

### NanoString analysis

Total RNA was collected from *P. aeruginosa* colony biofilms grown on MOPS-glucose agar with 0.7 mM K_2_HPO_4_/KH_2_PO_4_. Bacteria were grown overnight in LB, then sub-cultured in LB until OD_600_ = 0.5 before 5 µL was spotted on MOPS-glucose agar, and incubated at 37°C for 12 hours. Cells were then scraped from the agar and resuspended in 200 µL Tris-EDTA buffer. RNA was extracted using the Qiagen RNeasy kit (Qiagen 74004) using the protocol provided by the manufacturer. Samples were not treated with DNase. NanoString analysis of 80 ng of isolated RNA using codeset PaV5 (21) was performed as previously reported (81). Counts were normalized to the geometric mean of six housekeeping genes (*ppiD*, *rpoD*, *soj*, *dnaN*, *pepP*, and *dapF*). Normalized counts were used for heatmap construction.

### Growth curves

*P. aeruginosa* was grown in 5 mL LB medium overnight. A volume of 15–30 µL of the culture was pelleted, and the spent LB was removed prior to normalization in MOPS minimal medium with 20 mM glucose and no Pi to OD_600_ = 0.05. Then, 150 µL of cell culture was added to each well of a 96-well microtiter plate in triplicate. The wells surrounding the cultures were filled with water to mitigate edge effects. The plate was incubated in a Synergy Neo2 multimode plate reader with the lid and continuous shaking at 37°C. The OD_600_ was measured every 2 hours.

### Statistical analysis and figure design

Statistical analyses were performed and graphs were designed using GraphPad Prism version 10.3 for macOS. Fig. 4A and 7 were created in BioRender (BioRender.com/y62m090).

## SUPPLEMENTAL MATERIAL

File S1 jb.00189-24-s0001.xlsx NanoString data.

Supplemental figures and table jb.00189-24-s0002.pdf Figures S1 to S6 and Table 1.

ASM does not own the copyrights to Supplemental Material that may be linked to, or accessed through, an article. The authors have granted ASM a non-exclusive, world-wide license to publish the Supplemental Material files. Please contact the corresponding author directly for reuse.
