# Supplementary material for: Loss of LasR function leads to decreased repression of Pseudomonas aeruginosa PhoB activity at physiological phosphate concentrations
Source: J Bacteriol. 2025 May 14;207(6):e00189-24. doi: 10.1128/jb.00189-24 (PMC12186492; doi:10.1128/jb.00189-24)
Supplement: Supplemental figures and table — Figures S1 to S6 and Table 1. [file jb.00189-24-s0002.pdf]

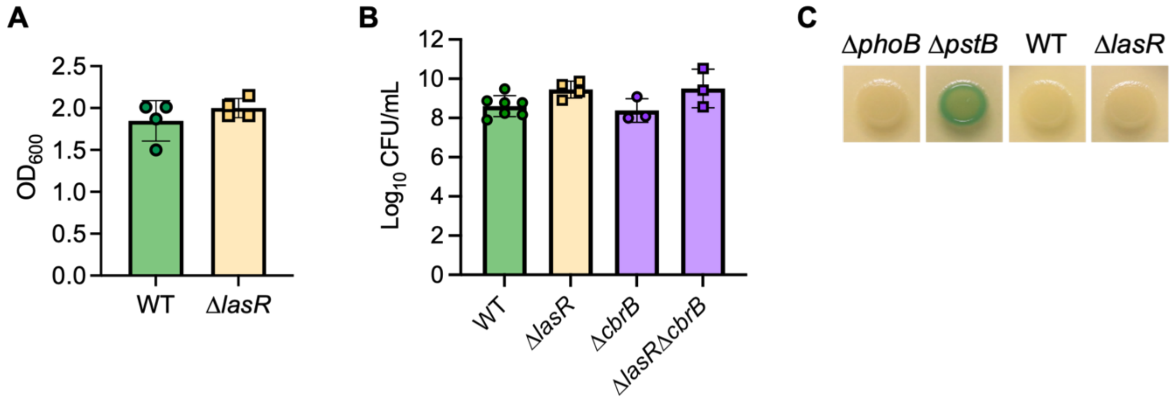

Fig S1 *P. aeruginosa* growth at 0.7 mM Pi and PhoB activity on LB agar. A) *P. aeruginosa* was grown in MOPS-glucose liquid medium with 0.7 mM Pi for 16 h at 37 °C. Data were analyzed using an unpaired t-test ($P = 0.3$, $n = 4$). **B)** *P. aeruginosa* was grown as a spot colony on MOPS-glucose agar with 0.7 mM Pi followed by enumeration of CFUs of the colony after resuspension in buffer as described in the *Methods*. Data were analyzed using an unpaired, ordinary one-way ANOVA with Tukey's multiple comparisons tests ($P > 0.15$ for all comparisons, $n = 3$-6). **C)** Colony biofilms of WT, Δ*phoB,* Δ*pstB,* and Δ*lasR* *P. aeruginosa* were grown on LB agar with 60 µg/mL BCIP. Similar results were obtained in three replicate experiments, and a representative image is shown above.

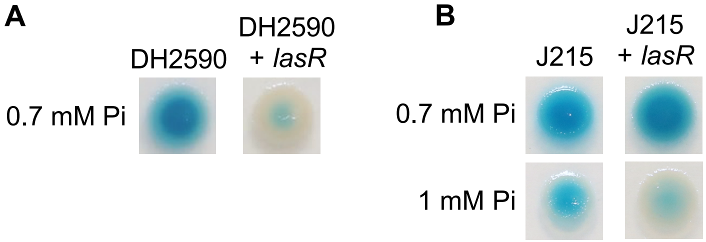

Fig S2 PhoB activity in LasR- clinical isolates. A-B) *P. aeruginosa* grown on MOPS-glucose agar with BCIP. **A**) DH2590 is a clinical isolate with a loss-of-function mutation in *lasR*. **B**) J215 is a clinical isolate with loss-of-function mutations in *lasR* and *rhlI*.

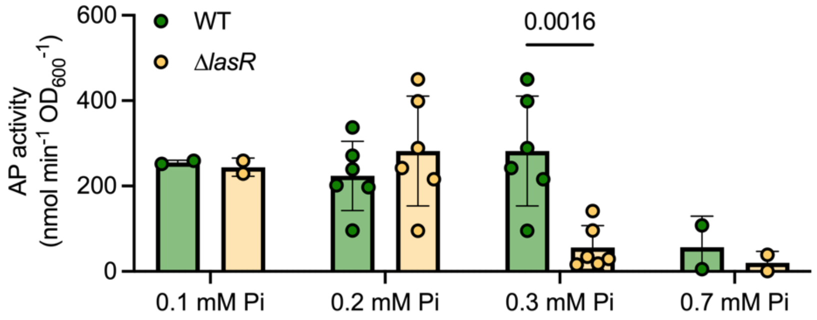

Fig S3. PhoB activity in liquid cultures. AP activity from *P. aeruginosa* grown at 21% oxygen in MOPS-glucose liquid medium for 12 h with varying Pi concentrations. Data were analyzed using an unpaired two-way ANOVA with Sidak's multiple comparisons tests. AP activity is significantly lower in Δ*lasR* at 0.3 mM Pi. There is no significant difference between WT and the Δ*lasR* mutant at the other Pi concentrations ($P > 0.7$, $n = 2$-6).

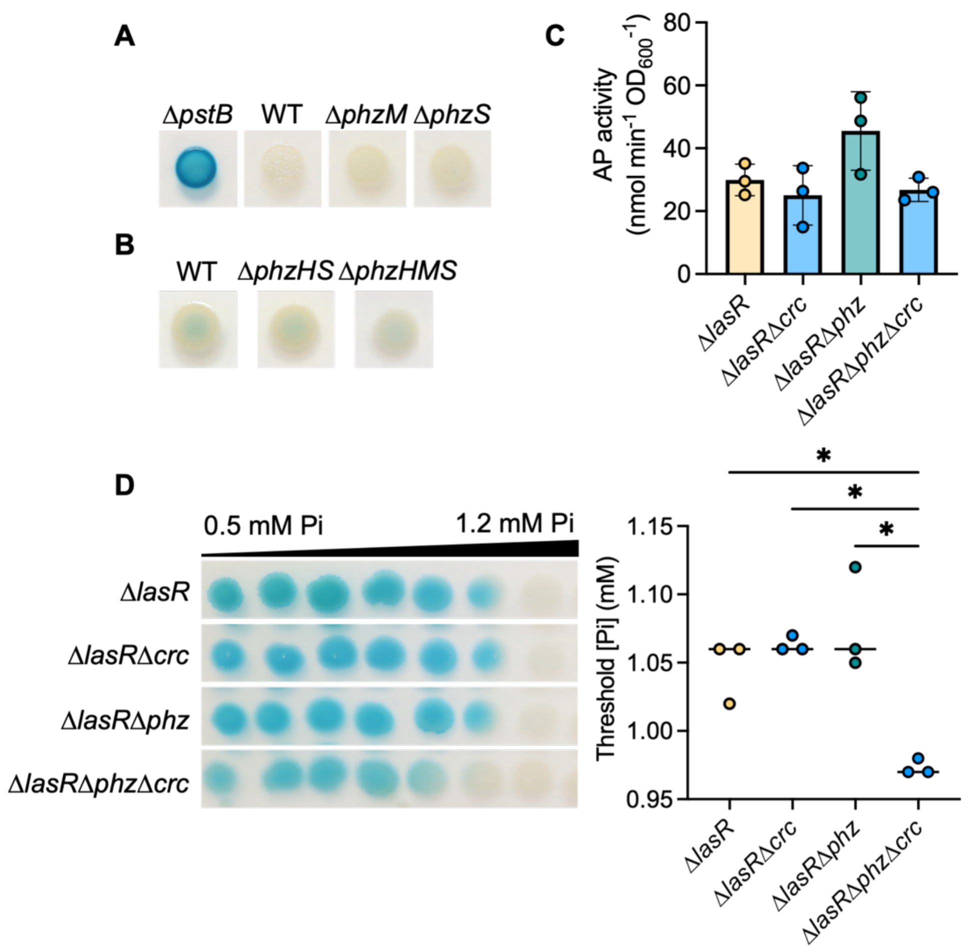

Fig S4. PhoB activity in phenazine mutants. A) Colony biofilms of the WT, *ΔpstB, ΔphzM, ΔphzS* mutants grown on MOPS-glucose agar with 0.7 mM Pi and 60 µg/mL BCIP. Similar results were obtained in three replicate experiments, and a representative image is shown above. **B)** *P. aeruginosa* WT, *ΔphzHS,* and *ΔphzHMS* mutants were grown as described in **A**. **C)** *P. aeruginosa* colony biofilms were grown on MOPS-glucose agar with 0.7 mM Pi for analysis of AP activity using the colorimetric PNPP substrate. Data were analyzed using a one-way ANOVA and Tukey's multiple comparisons tests. There were no significant differences between strains ($P \geq 0.07$, $n = 3$). **D**) *P. aeruginosa* was grown on MOPS-glucose agar with 60 µg/mL BCIP and a gradient of Pi (0.5 – 1.2 mM). The average concentration of Pi that inhibits AP activity is graphed to the right ($n = 3$). Data were analyzed using a one-way ANOVA. Asterisks denote significance ($P < 0.05$ = *).

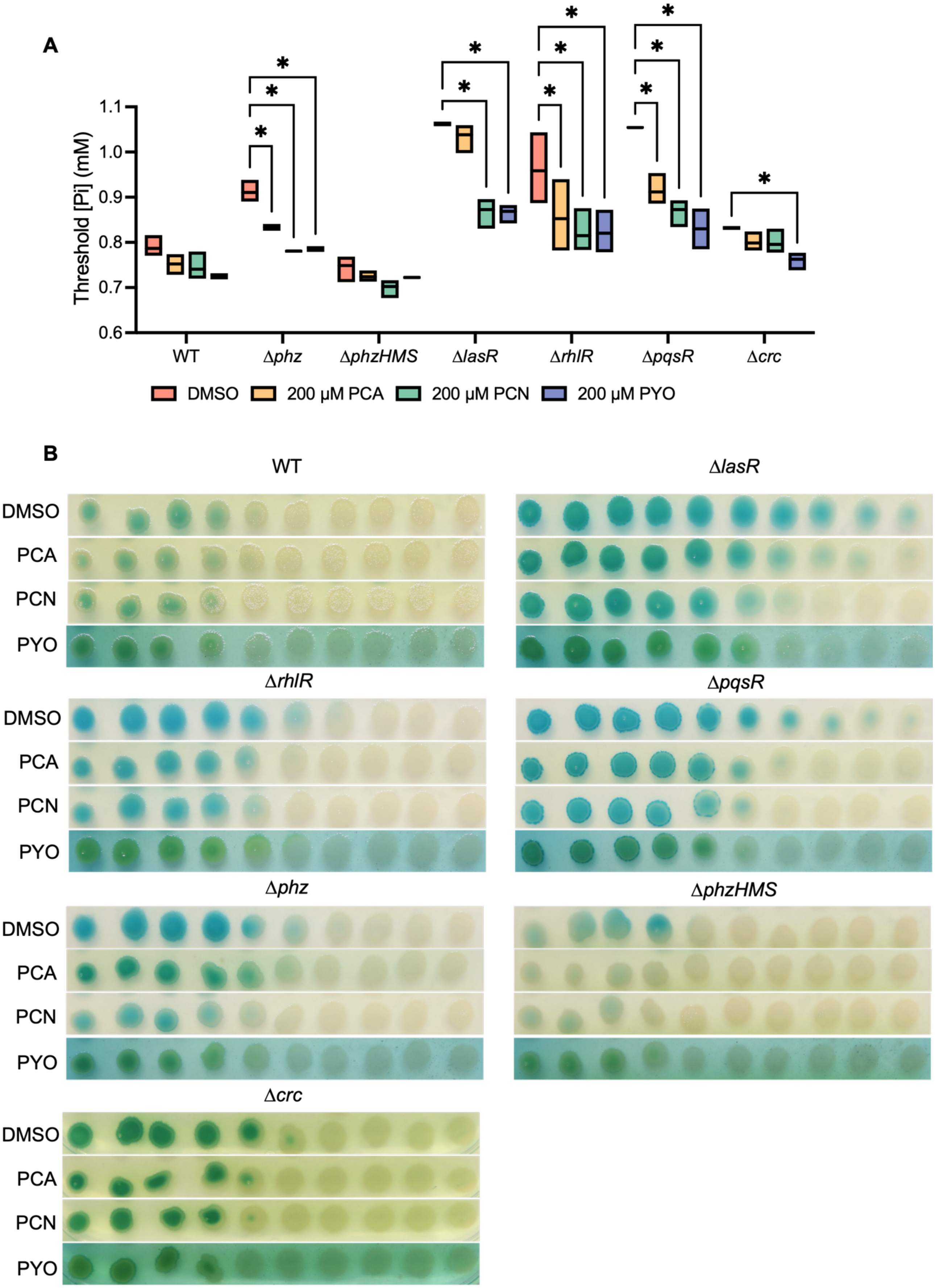

Fig S5. Phenazines alter threshold Pi for PhoB activity. *P. aeruginosa* was grown on MOPS-glucose agar with BCIP and a range of Pi concentrations (0.5 – 1.1 mM) and 200 µM of PCA, PCN, PYO, or vehicle control (DMSO). **A)** The Pi threshold was determined as described in the *Methods*. Data were analyzed using an unpaired two-way ANOVA and Dunnett's multiple comparisons tests (n = 3). Asterisks denote significance ($P < 0.05$ = *). **B**) Representative images of gradient Pi plates with BCIP.

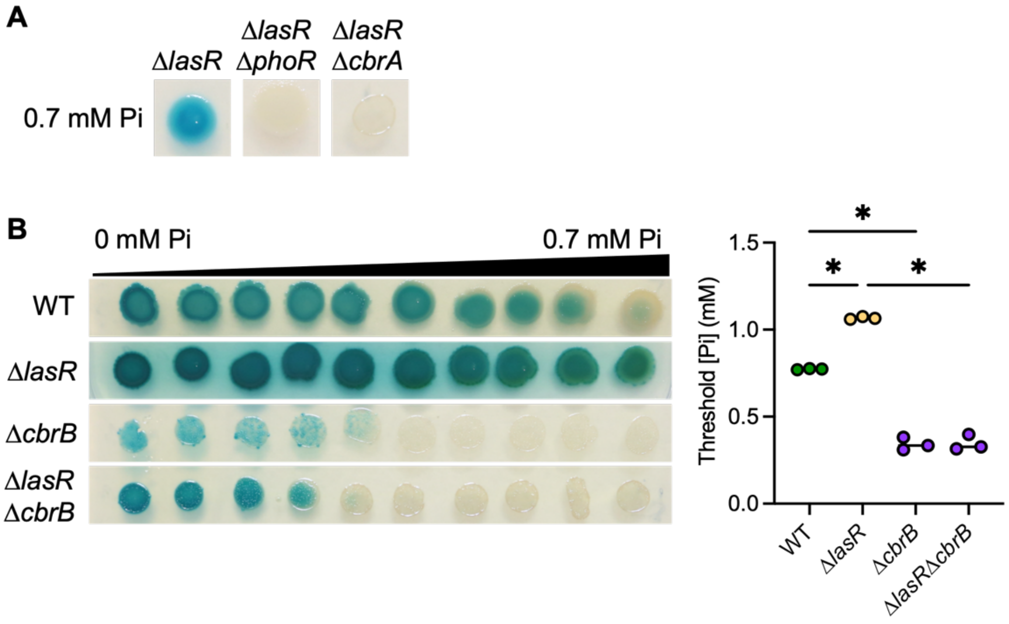

Fig S6. AP activity in Δ*cbrA* and Δ*cbrB* mutants. A) *lasR* was deleted from the PA14 kinase collection parent strain and mutants. Strains were grown on MOPS-glucose agar with BCIP and 0.7 mM Pi. **B)** *P. aeruginosa* was grown on MOPS-glucose agar with BCIP and a range of 0 – 0.7 mM Pi or 0.5 – 1.5 mM Pi (not pictured). The Pi threshold was determined as described in the *Methods*. Data were analyzed using a one-way ANOVA and Tukey's multiple comparisons tests (n = 3). Asterisks denote significance ($P < 0.05$ = *). There was no significant difference between Δ*cbrB* and Δ*lasR*Δ*cbrB* (P = 0.99, n = 3).

Supplemental Table 1. Strains and plasmids used in this study.

Strain	Strain ID	Description	Source
<u>*P. aeruginosa*</u>			
PA14 WT	DH122	Laboratory reference strain	(1)
PA14 Δ*lasR*	DH164	DH122 with in-frame deletion of *lasR*	(2)
PA14 Δ*lasR* + *lasR*	DH4740	SMC5021 with complementation of *lasR* at the native locus	(3)
PA14 Δ*phoB*	DH3284	DH122 with in-frame deletion of *phoB*	(4)
PA14 Δ*lasR*Δ*phoB*	DH4264	DH3284 with in-frame deletion of *lasR*	This study
PA14 Δ*pstB*	DH3601	DH122 with in-frame deletion of *pstB*	(4)
Clinical Isolate Pair 1 (LasR+)	DH1133	Clinical strain AMT0047-2. CF lung isolate with functional LasR.	(5)
Clinical Isolate Pair 1 (LasR-)	DH1132	Clinical strain AMT0047-3. CF lung isolate with loss-of-function mutation to *lasR*.	(5)
Clinical Isolate Pair 2 (LasR+)	DH2417	Clinical strain NC-AMT0101-2. CF lung isolate with functional LasR.	(5)
Clinical Isolate Pair 2 (LasR-)	DH2415	Clinical strain NC-AMT0101-1. CF lung isolate with loss-of-function mutation to *lasR.*	(5)
DH2590	DH2590	Clinical isolate from ocular infection with a mutation encoding $LasR^{I215S}$.	(6)
DH2590 + *lasR*	DH2743	Clinical Isolate DH2590 with complementation of functional *lasR* at the *lasR* locus	(8)
J215	DH2403	CF tracheal isolate with loss-of-function mutations to *lasR* and *rhlI.*	(9)
J215 + *lasR*	DH4584	DH2403 with PA14 *lasR* complemented at the *lasR* locus.	This study
PA14 Δ*lasR*	DH4208	DH2455 (7) with in-frame deletion of *lasR*	This study
PA14 Δ*lasR*Δ*cbrA*	DH4249	DH2507 (7) with in-frame deletion of *lasR*	This study
PA14 Δ*lasR*Δ*phoR*	DH4255	DH2516 (7) with in-frame deletion of *lasR*	This study
PA14 Δ*rhlR*	DH2742	DH122 with in-frame deletion of *rhlR*	(10)
PA14 Δ*lasR*Δ*rhlR*	DH2944	DH164 with in-frame deletion of *rhlR*	(10)
PA14 Δ*pqsR*	DH1110	DH122 with in-frame deletion of *pqsR*	(11)
PA14 Δ*lasR*Δ*pqsR*	DH1111	DH164 with in-frame deletion of *pqsR*	(12)
PA14 Δ*phz1*	DH1728	DH122 with in-frame deletion of *phzA1-G1* operon	(13)
PA14 Δ*phz2*	DH1735	DH122 with in-frame deletion of *phzA2-G2* operon	(13)

PA14 Δ*phz*	DH933	PA14 with in-frame deletions of both *phzA1-G1* and *phzA2-G2* operons	(13)
PA14 Δ*lasR*Δ*phz*	DH4501	In-frame deletions of *phzA1-G1*, *phzA2-G2,* and *lasR*	(3)
PA14 Δ*phzHS*	DH4570	In-frame deletions of *phzH* and *phzS*	(14)
PA14 Δ*phzHMS*	DH4572	In-frame deletions of *phzH, phzM,* and *phzS*	(15)
PA14 Δ*phzM*	DH1774	In-frame deletion of *phzM*	(16)
PA14 Δ*phzS*	DH1393	In-frame deletion of *phzS*	(16)
PA14 Δ*cbrB*	DH3920	DH122 with in-frame deletion of *cbrB*	(17)
PA14 Δ*lasR*Δ*cbrB*	DH3924	DH164 with in-frame deletion of *cbrB*	(17)
PA14 Δ*lasR*Δ*cbrB* + *cbrB*	DH3925	DH3924 with complementation of *cbrB* at the native locus	(17)
PA14 Δ*crc*	DH3737	DH122 with in-frame deletion of *crc*	(17)
PA14 Δ*crc* + *crc*	DH3738	DH3737 with complementation of *crc* at the native locus	(17)
PA14 Δ*lasR*Δ*crc*	DH3927	DH164 with in-frame deletion of *crc*	(17)
PA14 Δ*lasR*Δ*cbrB*Δ*crc*	DH3926	DH3924 with in-frame deletion of *crc*	(17)
PA14 Δ*phz*Δ*crc*	DH4174	DH933 with in-frame deletion of *crc*	This study
PA14 Δ*lasR*Δ*phz*Δ*crc*	DH4563	DH4174 with in-frame deletion of *lasR*	This study
E. coli			
S17 λpir	DH71	Used as a conjugation partner for introducing pMQ30-based plasmids	
DH5α	DH51	Used to store/replicate plasmids	Invitrogen
Plasmids			
pMQ30 EV	DH962	Allelic replacement vector for use in yeast cloning, GmR	(18)
pMQ30_*lasR*_KO	DH2918	*lasR* in-frame deletion construct, GmR	(2)
pMQ30_*lasR*	DH3548	*lasR* in-frame complementation construct, GmR	(19)
pMQ30_*crc*_KO	DH3692	*crc* in-frame deletion construct, GmR	(17)

1. Rahme LG, Tan M-W, Le L, Wong SM, Tompkins RG, Calderwood SB, Ausubel FM. 1997. Use of model plant hosts to identify *Pseudomonas aeruginosa* virulence factors. Proceedings of the National Academy of Sciences 94:13245-13250.
2. Hogan DA, Vik Å, Kolter R. 2004. A *Pseudomonas aeruginosa* quorum-sensing molecule influences *Candida albicans* morphology. Molecular Microbiology 54:1212-1223.
3. Jean-Pierre F, Hampton TH, Schultz D, Hogan DA, Groleau M-C, Déziel E, O'Toole GA. 2023. Community composition shapes microbial-specific phenotypes in a cystic fibrosis polymicrobial model system. eLife 12:e81604.
4. Doing G, Koeppen K, Occipinti P, Harty CE, Hogan DA. 2020. Conditional antagonism in co-cultures of *Pseudomonas aeruginosa* and *Candida albicans*: an intersection of ethanol and phosphate signaling distilled from dual-seq transcriptomics. PLoS genetics 16:e1008783.
5. Smith EE, Buckley DG, Wu Z, Saenphimmachak C, Hoffman LR, D'Argenio DA, Miller SI, Ramsey BW, Speert DP, Moskowitz SM, Burns JL, Kaul R, Olson MV. 2006. Genetic adaptation by *Pseudomonas aeruginosa* to the airways of cystic fibrosis patients. Proceedings of the National Academy of Sciences 103:8487-8492.
6. Hammond JH, Hebert WP, Naimie A, Ray K, Van Gelder RD, DiGiandomenico A, Lalitha P, Srinivasan M, Acharya NR, Lietman T, Hogan DA, Zegans ME. 2016. Environmentally endemic *Pseudomonas aeruginosa* strains with mutations in *lasR* are associated with increased disease severity in corneal ulcers. mSphere 1:00140-16.
7. Wang BX, Cady KC, Oyarce GC, Ribbeck K, Laub MT. 2021. Two-component signaling systems regulate diverse virulence-associated traits in *Pseudomonas aeruginosa*. Applied and environmental microbiology 87:e03089-20.
8. Clay ME, Hammond JH, Zhong F, Chen X, Kowalski CH, Lee AJ, Porter MS, Hampton TH, Greene CS, Pletneva EV, Hogan DA. 2020. *Pseudomonas aeruginosa lasR* mutant fitness in microoxia is supported by an Anr-regulated oxygen-binding hemerythrin. Proceedings of the National Academy of Sciences 117:3167-3173.
9. Hammond JH, Dolben EF, Smith TJ, Bhuju S, Hogan DA. 2015. Links between Anr and quorum sensing in *Pseudomonas aeruginosa* biofilms. Journal of Bacteriology 197:2810-2820.
10. Harty CE, Martins D, Doing G, Mould DL, Clay ME, Occhipinti P, Nguyen D, Hogan DA. 2019. Ethanol stimulates trehalose production through a SpoT-DksA-AlgU-Dependent pathway in *Pseudomonas aeruginosa*. Journal of Bacteriology 201:e00794-18.
11. Cugini C, Morales DK, Hogan DA. 2010. *Candida albicans*-produced farnesol stimulates *Pseudomonas* quinolone signal production in LasR-defective *Pseudomonas aeruginosa* strains. Microbiology 156:3096.
12. Mould DL, Botelho NJ, Hogan DA, Goldberg JB. 2020. Intraspecies signaling between common variants of *Pseudomonas aeruginosa* increases production of quorum-sensing-controlled virulence factors. mBio 11:e01865-20.
13. Dietrich LE, Price-Whelan A, Petersen A, Whiteley M, Newman DK. 2006. The phenazine pyocyanin is a terminal signalling factor in the quorum sensing network of *Pseudomonas aeruginosa*. Mol Microbiol 61:1308-21.
14. Bellin DL, Sakhtah H, Rosenstein JK, Levine PM, Thimot J, Emmett K, Dietrich LE, Shepard KL. 2014. Integrated circuit-based electrochemical sensor for spatially resolved detection of redox-active metabolites in biofilms. Nat Commun 5:3256.
15. Recinos DA, Sekedat MD, Hernandez A, Cohen TS, Sakhtah H, Prince AS, Price-Whelan A, Dietrich LE. 2012. Redundant phenazine operons in *Pseudomonas aeruginosa* exhibit environment-dependent expression and differential roles in pathogenicity. Proceedings of the National Academy of Sciences 109:19420-19425.
16. Sakhtah H, Koyama L, Zhang Y, Morales DK, Fields BL, Price-Whelan A, Hogan DA, Shepard K, Dietrich LEP. 2016. The *Pseudomonas aeruginosa* efflux pump MexGHI-OpmD transports a natural phenazine that controls gene expression and biofilm development. Proceedings of the National Academy of Sciences 113:E3538-E3547.

17. Mould DL, Stevanovic M, Ashare A, Schultz D, Hogan DA. 2022. Metabolic basis for the evolution of a common pathogenic *Pseudomonas aeruginosa* variant. eLife 11:e76555.
18. Shanks RM, Caiazza NC, Hinsa SM, Toutain CM, O'Toole GA. 2006. *Saccharomyces cerevisiae*-based molecular tool kit for manipulation of genes from gram-negative bacteria. Applied Environmental Microbiology 72:5027-36.
19. Clay ME, Hammond JH, Zhong F, Chen X, Kowalski CH, Lee AJ, Porter MS, Hampton TH, Greene CS, Pletneva EV. 2020. *Pseudomonas aeruginosa lasR* mutant fitness in microoxia is supported by an Anr-regulated oxygen-binding hemerythrin. Proceedings of the National Academy of Sciences 117:3167-3173.
